# Barriers and facilitators for social inclusion among people with concurrent mental health and substance use problems. A qualitative scoping review

**DOI:** 10.1371/journal.pone.0315758

**Published:** 2024-12-16

**Authors:** Silje Nord-Baade, Ottar Ness, Camilla Bergsve Jensen, Michael Rowe, Elin Opheim, Anne Landheim

**Affiliations:** 1 Norwegian National Advisory Unit on Concurrent Substance Abuse and Mental Health Disorders, Innlandet Hospital Trust, Hamar, Norway; 2 Inland Norway University of Applied Sciences, Elverum, Norway; 3 Norwegian University of Science and Technology, Trondheim, Norway; 4 School of Medicine, Yale University, New Haven, Connecticut, United States of America; University of Foggia: Universita degli Studi di Foggia, ITALY

## Abstract

**Background:**

People with concurrent mental health and substance use problems are among the most socially excluded groups in our society, yet little attention has been paid to what socially excluded people see as central to promoting their social inclusion. The aim of this qualitative scoping review is to provide an overview of barriers and facilitators for social inclusion among people with concurrent mental health and substance use problems, based on first-person perspectives, to help guide future research, policies, and practice.

**Methods:**

We explored first-person perspectives on social inclusion among people with concurrent mental health and substance use problems, employing Arksey and O’Malleys framework. We searched Medline, PsycINFO, Embase, Scopus, Cinahl, and other sources for studies published between January 2000 and September 2023. We employed content analysis and followed the PRISMA checklist.

**Results:**

We included 55 articles included in our review and identified sub themes of: Intrapersonal baseline (identity, belonging), Components of social inclusion (relationships, meaningful activities, employment, economy), and Systemic failure or success (housing, public health and welfare services, the criminal justice system).

**Conclusion:**

Social inclusion is rarely studied outside the context of direct services. Our results point to knowledge gaps in addressing social inclusion in a broad, societal context; implementing gaps in services; and developing policies to assure the fundamental needs and human rights of socially excluded persons.

## Introduction

People with concurrent mental health and substance use problems are among the most socially excluded groups in our society [1, 2]. In short, addressing social inclusion is essential to promote their well-being and quality of life [1]. Measuring the prevalence of dual disorders, or concurrent mental health and substance use disorders, is a complicated matter. One study found concurrent substance use disorders in approximately 30% of all patients diagnosed with severe mental illness [3]. Another study reported a lifetime prevalence of a psychiatric disorder in up to 89% of people with substance use disorders [4]. The complexity of these disorders and their prevalence supports the need to address marginalization in this group, with probability of providing transferable knowledge to other marginalized groups such as immigrants and refugees, people with LGBTQ+ sexual orientations, and people with other physical or mental disabilities [5].

Social exclusion has been defined as “a state in which individuals are unable to participate fully in economic, social, political and cultural life, as well as the process leading to and sustaining such a state” [5 p. 18]. It is linked to mental and physical illness, lower quality of life, and higher morbidity and mortality [6]. We endorse an understanding of social inclusion as both a process and a goal, that is, as “the process of improving the terms of participation in society for people who are disadvantaged on the basis of age, sex, disability, race, ethnicity, origin, religion, or economic or other status, through enhanced opportunities, access to resources, voice and respect for rights” [5 p. 20]. These definitions provide a broad understanding of the concepts, enabling us to explore the complexity of the matter. However, the measurement and definitions of social inclusion and exclusion is dependent on systemic, social, cultural, material, subjective and psychological factors [7–9]. Addressing social inclusion among people with concurrent mental health and substance problems is especially important in a time of increasing social inequality and a difficult global economy that disproportionately affects those with limited resources. Organizations such as the United Nations [5] highlight the importance of addressing mental health and substance use issues and associated social inequity that may constitute violations of human rights.

Experiencing social inclusion (e.g. through relationships, activities, work, housing and a sense of belonging) facilitates recovery. It also has preventive effects against mental health problems and substance use via the experience of receiving social support, engaging and participating in activities, maintaining and developing social skills, and of a sense of belonging and empowerment [10, 11]. Yet despite acknowledging the benefits of social inclusion, service providers often overlook the community aspects of patients’ lives [12]. Even though the recovery approach has improved service quality, critiques of mental health and substance use services suggest an overemphasis on individual responsibility. Further, service providers are critiqued for not addressing structural factors and societal influences that function as barriers for social inclusion for the individual [10, 13]. In addition, efforts have been made to implement knowledge-based service models with social inclusion as an essential component. However, such models, e.g. Assertive Community Treatment Teams (ACT), Flexible Assertive Community Treatment (FACT), Individual Placement and Support (IPS), Integrated Dual Disorders Treatment (IDDT), and Housing First are not implemented systematically. This has leadto disparate treatments and services available to the target group [14–16].

When addressing social inclusion and exclusion, there is a tendency to focus on objective measures related to work, education, housing, and economy. The individuals’ subjective experiences of promoting social inclusion are understudied [9]. This study seeks to complement the knowledge on objective measures and emphasize the person-oriented approach, aimed at improving the services’ ability to promote social inclusion and inform future research and policies. The research question of this qualitative scoping review is: what are the barriers and facilitators for social inclusion as experienced by people with concurrent mental health and substance use problems?

## Materials and methods

A scoping review was chosen as it is suitable for broad, complex, and multidisciplinary research questions, and for exploring key characteristics or factors related to the research topic. It enables mapping of existing literature and knowledge gaps, hence providing guidance and directions for future research, practice and policy efforts [17–19]. The study employed Arksey and O´Malley’s [17] five-stage framework for scoping reviews: (1) identifying the research questions, (2) searching for relevant studies, (3) selecting studies, (4) charting the data, and (5) collating and summarizing the studies. In this section, the conduction of the first four phases is presented while covering the fifth stage in the results section, following the PRISMA checklist for scoping reviews [20] (see S1 Checklist).

### Stage 1 –Identifying the research question

Little is known about promoting social inclusion based on the experience of marginalized groups, including people with concurrent mental health and substance use problems. Focusing on the voices of this group can support their empowerment and help us bridge the gap between research and practice. The aim in this study was to present current knowledge status regarding the question: *What are the barriers and facilitators for social inclusion as experienced by people with concurrent mental health and substance use problems*?

### Stage 2 –Searching for relevant studies

**Fig 1:**
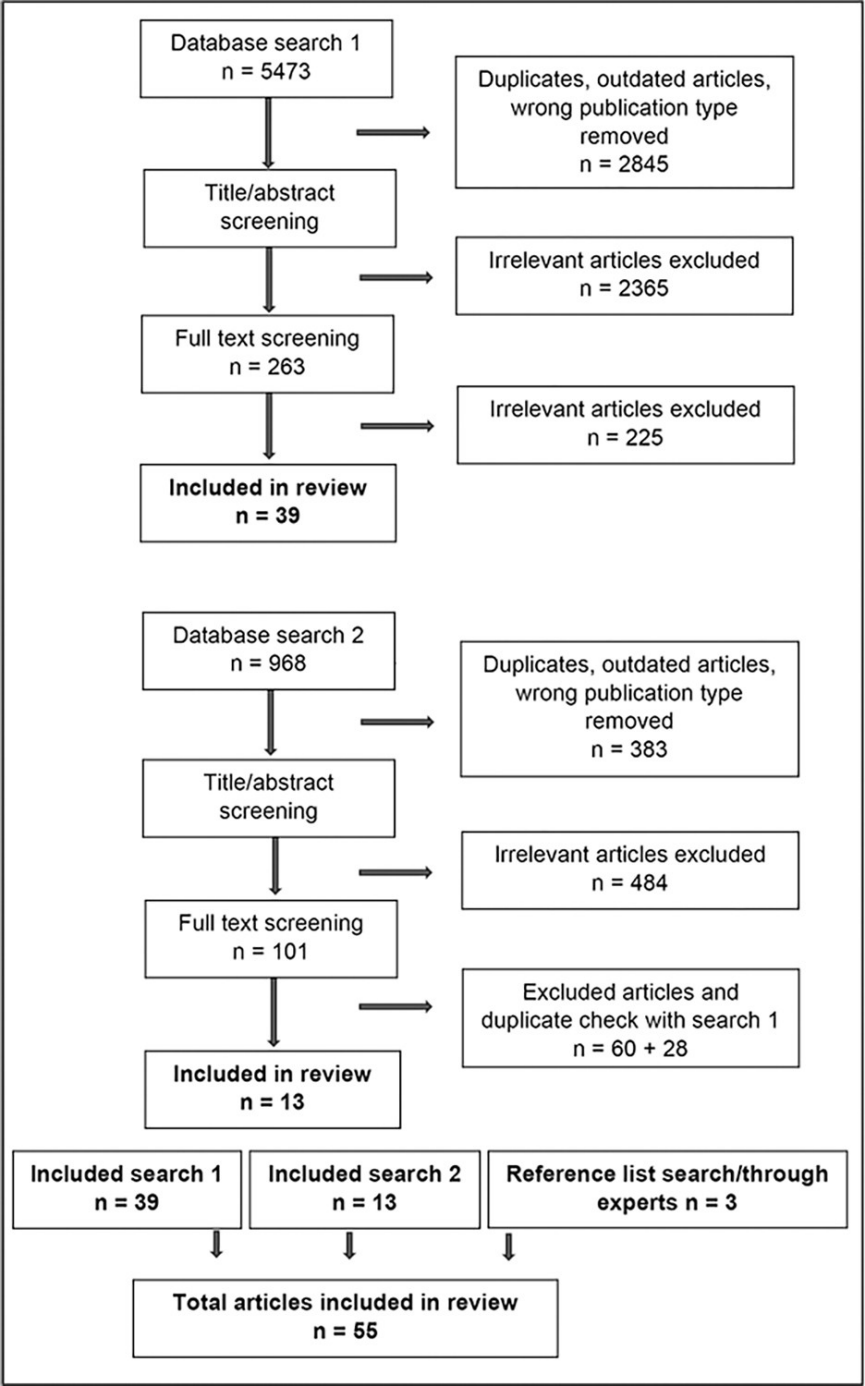
Scoping review search strategy.

The search was conducted in two phases (see S1 File and Fig 1). First, SNB and EO conducted a broad exploratory search for both quantitative and qualitative research through Medline (OVID), PsycINFO (OVID), Embase (OVID), Scopus (Elsevier) and Cinahl (Ebsco) in February and March 2023. After screening the results, discussions among all authors led to a decision to include articles with first-person perspectives only, as these best reflected the wisdom that persons in the target group could offer. Second, in September 2023, EO conducted a search in the same databases as above, including the following text words and subject terms, aimed at discovering research with first-person perspectives: (1) concurrent mental health and substance use, (2) mental illness in combination with (3) substance/alcohol use, (4) social inclusion, and (5) first-person experiences (second search only). Third, supplementary searches were conducted through searching reference lists manually and contacting experts in the field to identify additional studies. The supplementary search uncovered few references that the database search had not.

### Stage 3 –Selecting studies

SNB and ON developed a screening guide that included qualitative peer reviewed empirical research articles with first-person experiences of social inclusion in the target group, published in the years 2000–2023, and written in English or Scandinavian languages (Norwegian, Swedish, Danish). Included articles originated in similar countries according to criteria outlined by Esping Andersen [21] and Bambra [22]. All Nordic countries were included. Exclusion criteria in addition to not meeting the inclusion criteria were articles not addressing barriers and facilitators for social inclusion.

#### Eligibility criteria

2628 articles were screened by title, abstracts, and keywords in the first phase. Of these, 263 met criteria for full text screening, and of these 39 were included in the final analysis. In a second search 585 articles were screened by title, abstracts, and keywords. 101 qualified for full text screening; of these, 42 articles met the inclusion criteria; and of these, 13 articles were included after de-deduplicating articles from the first search using EndNote. A total of 52 articles were included from the searches. Three additional articles were retrieved from reference lists and through experts, leading to a total of 55 articles for the scoping review (see Fig 1).

SNB and CBJ, a peer researcher, read all abstracts in the first search (n = 2628). In this phase, studies that did not highlight the target group were included because they included relevant information on, or discussion of high numbers of concurrent mental health and substance use problems. Studies that noted but did not highlight social inclusion, focusing instead on recovery or other themes, were also included. Non-empirical articles were excluded, and those that fell under other exclusion criteria. Several other articles were excluded in this phase, often connected to the search words ‘co-occurring’ and ‘concurrent’. This led the databases to include studies on other comorbid conditions and diagnoses in the results. Consensus was reached on included articles for full text screening after consulting with ON. SNB read 100% of the articles included for full text screening (n = 263). MR and AL each read 20% and 10% of the articles randomly selected. In the second search, SNB read all abstracts (n = 585) and conducted the full text screening (n = 101), following the same principles as described above. Of the finally included articles (n = 55), ON, MR and AL read 10 percent each, randomly.

### Stage 4 –Charting the data

SNB coded included articles by author, year published, title, journal, country of origin, methods, context and sample, main research focus, and subthemes (see Tables 1 and 2).

**Table 1:** Main themes and subthemes.

Main theme	Subtheme			
The intrapersonal baseline	Identity	Belonging		
Components of social inclusion	Relationships	Meaningful activities	Employment	Economy
Systemic failure or success	Housing	Public health and welfare services	The criminal justice system	

**Table 2:** Included articles and results.

Author(s), year published (reference number), title, journal	Country of origin	Method	Context and sample	Main research aim	Outcomes
					Extracted data by subthemes
Asher & Gask, 2010 [26] Reasons for illicit drug use in people with schizophrenia: Qualitative study, BMC Psychiatry, 2010	England	Individual interviews	17 people with schizophrenia and history of substance use. Recruited through local psychiatric services. 16 male, one female, ages from 16 and up, majority (n = 10) in ages 35–39, all living in a city area.	Exploring reasons for illicit drug use among people with schizophrenia	Identity Belonging Relationships Meaningful activities Employment Housing
Bauld et al., 2013 [27] Pathways back to work for problem alcohol users, Policy Studies, 2013	England, Scotland and Wales	Individual interviews, as part of a bigger study with systematic literature review and interviews with professionals.	53 treatment service clients currently accessing treatment for an alcohol problem. The majority had concurrent mental health problems. 38 male, 15 female, ages from 22–63, living in semi-rural and urban areas.	Exploring issues related to employment, unemployment, and alcohol misuse.	Identity Employment Economy
Beynon et al., 2009 [28] Self-reported health status, and health service contact, of illicit drug users aged 50 and over: A qualitative interview study in Merseyside, United Kingdom, BMC Geriatrics, 2009	England	Semi-structured interviews	Ten people with substance use problems, nine male, one female, ages 54–61 years, living in urban areas. The majority had concurrent mental health problems. Recruited through substance use treatment services.	Identify substance (drug and alcohol) use, self-reported health status and contact with generic and specialist health services of a cohort of older drug users in contact with specialist drug treatment services.	Relationships Public health and welfare services
Blank et al., 2016 [29] The lived experience of people with mental health and substance misuse problems: Dimensions of belonging, British Journal of Occupational Therapy	England	Individual interviews	Four people over the age of 18 with concurrent mental health and substance use problems recruited through online advertisement. Additional information on sample and context is not disclosed.	Exploring the meaning and experience of belonging for people with dual diagnosis.	Identity Belonging Relationships Housing
Brekke et al., 2017 [30] First-person experiences of recovery in co-occurring mental health and substance use conditions, Advances in Dual Diagnosis	Norway	In-depth individual interviews	Eight persons with concurrent conditions receiving treatment from a mental health and addictions team. Four male, four female, ages from early twenties to seventies, in rural areas. Recruited by flyer handouts through services, a peer support house, local NA group and low-threshold meeting places.	Explore and describe experiences of recovery among people with concurrent mental health and substance use conditions in a rural community in Norway.	Identity Belonging Relationships Meaningful activities Employment Economy Housing
Brekke et al., 2020 [31] Relational recovery in co-occurring conditions: a qualitative study of first-person experiences, Advances in Dual Diagnosis	Norway	In-depth individual interviews	Eight persons with concurrent conditions receiving treatment from a mental health and addictions team. Four male, four female, ages from early twenties to seventies, in rural areas. Recruited by flyer handouts through services, a peer support house, local NA group and low-threshold meeting places.	Explore and describe first-person experiences of relational recovery in concurrent mental health and substance use conditions.	Relationships Employment
Brekke et al., 2021 [32] Service User Experiences of How Flexible Assertive Community Treatment May Support or Inhibit Citizenship: A Qualitative Study, Frontiers in Psychology	Norway	In-depth individual interviews	32 service users from five Norwegian FACT teams, where 20 participants reported current or previous concurrent mental health and substance use problems. 21 male, 11 female, from ages 20–67, mean age 37, living in both urban and rural areas.	The aim of this study was to explore and describe service user experiences of how receiving services from a Flexible Assertive Community Treatment (FACT) team may support or inhibit citizenship.	Identity Belonging Relationships Employment Economy Housing Public health and welfare services
Brooks et al., 2007 [33] Consumers perspectives on co-occurring disorders treatment, Journal of Drug Issues	USA	Focus group interviews	35 people with a concurrent mental illness and substance use problems recruited through receiving treatment as usual (n = 24) and the ADMIRE Plus program (n = 11), an intensive day treatment program. 23 males, 22 females, that had received treatment services 7,5 years on average (range three months– 25 years)	Describe the treatment experiences and identify factors facilitating and/or hindering treatment progress. A secondary purpose was to compare the responses of clients who received services through a totally integrated program versus a more fragmented approach in a traditional outpatient program with services coordinated by a case manager.	Identity Belonging Relationships Public health and welfare services The criminal justice system
Chilton et al., 2020 [34] ‘The group was the only therapy which supported my needs, because it helped me feel normal and I was able to speak out with a voice’: A qualitative study of an integrated group treatment for dual diagnosis service users within a community mental health setting, International Journal of Mental Health Nursing	England, Wales	Semi-structured interviews	15 people with concurrent mental health and drug problems, majority psychotic disorders and alcohol use disorders. Nine male, six female, ages 24–65, all had completed the PEG program.	Explore the individual experiences of participants receiving treatment from a integrated, psychoeducational group (PEG).	Identity Belonging Relationships Public health and welfare services
Chiringa et al., 2014 [35] Reasons for recall following conditional discharge: explanations given by male patients suffering from dual diagnosis in a London Forensic Unit, Journal of Psychiatric and Mental Health Nursing	England	Semi-structured interviews	Six people with concurrent mental health and drug problems, recalled and readmitted to forensic services following conditional discharge. All participants were male, mean age 46 years (range 35–57 years). The mean duration of stay in the community prior to recall was 4 and a half years, and the range was 1–11 years.	This study explores how male patients suffering from dual diagnosis in a forensic unit perceive being recalled and readmitted following conditional discharge and their views about how services might be improved.	The criminal justice system
Cruce et al., 2012 [36] Recovery-promoting Care as Experienced by Persons with Severe Mental Illness and Substance Misuse, International Journal of Mental Health and Addiction	Sweden	Individual in-depth interviews, each participant interviewed on two occasions.	Eight participants with psychotic disorders and concurrent misuse receiving treatment from an integrated outpatient program in an urban area. Six males, two females, age from 27–54 years.	Explore recovery-promoting care as experienced by persons with concomitant severe mental illness and substance misuse.	Identity Belonging Relationships Meaningful activities Housing Public health and welfare services
Davis & O’Neill, 2005 [37] A focus group analysis of relapse prevention strategies for persons with substance use and mental disorders, Psychiatric Services	USA	Focus group interviews	27 people in the later stages of substance abuse treatment recruited through a large psychosocial rehabilitation organization in a urban area. 12 females, 15 males, mean age 43.85, SD 8.77. 20 participants had a psychotic disorder, six had a major affective disorder, nine had a diagnosis of substance abuse, 18 a diagnosis of substance dependence.	Determine the strategies and supports persons with dual diagnoses rely on in their relapse prevention efforts.	Identity Relationships Meaningful activities Employment
De Ruysscher et al., 2019 [38] ‘A place to be (me)’: a qualitative study on an alternative approach to treatment for persons with dual diagnosis, Drugs: Education, Prevention and Policy	Belgium	In-depth interviews	Three participants (professionals and others interviewed excluded), one male, two female, age 41–60, with concurrent mental health and drug problems. All visitors at a community-based meeting center in a large city.	Gain insight into an alternative community-based approach to treatment for persons with dual diagnosis, by unraveling the daily practice of Villa Voortman from an idiographic qualitative perspective.	Identity Belonging
EnglandKennedy & Horton, 2011 [39] "Everything that I thought that they would be, they weren’t:" family systems as support and impediment to recovery, Social Science & Medicine	USA	Verbally administered structured and semi-structured interviews	122 people with COD recruited from 14 behavioral health agencies, including community mental health centers, residential and outpatient treatment centers, and small group practices, located in six counties, three rural and three with large urban areas. (Family members excluded in data collection).	Examine family help provision for adults diagnosed with concurrent severe mental illness and substance dependence.	Identity Belonging Relationships
Green et al., 2015 [40] Dual Recovery among People with Serious Mental Illnesses and Substance Problems: A Qualitative Analysis, Journal of Dual Diagnosis	USA	Part of a larger exploratory mixed methods study. In-depth interviews conducted with each participant over two years (2 at baseline, 1 each at 1- and 2-year follow-ups).	177 members of Kaiser Permanente Northwest (a group-model, not-for-profit, integrated health plan) with concurrent mental health and substance use problems. Age from 16–84 years (M = 48.8), 52% females.	Exploring individuals’ perspectives regarding their dual recovery experience.	Relationships
Haskell et al., 2016 [41] Service user and family member perspectives on services for mental health, substance use/addiction, and violence: a qualitative study of their goals, experiences and recommendations, International Journal of Mental Health Systems	Canada	Structured interviews	73 service users in two Ontario communities (one urban, one rural). Recruited through posters and flyers in mental health, addiction, and violence agencies, social service agencies, community organizations, public locations, and in local newspapers. 36 males, 37 females, adult population.	Explore experiences related to help-seeking and service user goals, positive and negative experiences, and recommendations for improving systems of care	Relationships Meaningful activities Employment Public health and welfare services
Hawkins & Abrams, 2007 [42] Disappearing acts: The social networks of formerly homeless individuals with co-occurring disorders, Social Science & Medicine	USA	72 in-depth qualitative interviews with 39 participants. 33 completed two interviews.	39 formerly homeless mentally ill individuals, living in an urban area. The majority with long term substance abuse. 33 completed two interviews. 67% males, 33% females, age range 23–62 (M = 48). Purposive sampling from previous study, New York Services Study.	Examine reasons for small social networks of individuals with concurrent disorders, and the impact of small networks for this population.	Relationships
Henwood et al., 2012 [43] Substance Abuse Recovery after Experiencing Homelessness and Mental Illness: Case Studies of Change Over Time, Journal of Dual Diagnosis	USA	In-depth interviews and used case study analysis	38 individuals who met criteria for having achieved a measure of success in mental health recovery, purposively sampled from two supportive housing agencies, one using a harm reduction and the other an abstinence model. Urban area, 26 males, 5 females, mean age 51.	To examine what can be learned about substance abuse recovery from consumers considered to be doing well; how past substance abuse fits into their present-day narratives; and how (if at all) policies of harm reduction versus abstinence are regarded as affecting recovery efforts.	Relationships Housing
Henwood et al., 2015 [44] Social Relationships of Dually Diagnosed Homeless Adults Following Enrollment in Housing First or Traditional Treatment Services, Journal of the Society for Social Work and Research	USA	Mixed-methods social network analysis approach. 42 participants were interviewed to contextualize quantitative findings.	A subset of 42 participants from a sample of 75 (males 51, females 24, age M = 41.28, SD 10.18). Dual-diagnosed homeless adults offered treatment, housing, or both. 15 from HF program, 15 from TF program, 12 participants that had disengaged. Urban area.	This study examines differences in the social networks of participants newly enrolled in programs that use either a Housing First (HF) approach (i.e., provides immediate access to permanent housing with ongoing consumer-driven support services) or a treatment first (TF) approach (i.e., traditional clinician-driven staircase model that requires temporary or transitional housing and treatment placements before accessing permanent housing).	Relationships Housing
Hodgson et al., 2001 [45] The Leisure Participation of Clients with a Dual Diagnosis, British Journal of Occupational Therapy	Australia	In-depth, semi-structured interviews.	Four outpatients from an alcohol and drug rehabilitation program. Age 22–32 years, three males, one female.	Explore the leisure participation of clients with a dual diagnosis.	Relationships Meaningful activities Economy
Johnson et al., 2013 [46] “I know if I drink I won’t feel anything”: Substance use relapse among depressed women leaving prison, International Journal of Prisoner Health	USA	Semi-structured interviews conducted after prison release	15 women with concurrent substance use and major depressive disorders. Mean age 39, SD 9.4.	Explore treatment needs and factors contributing to engagement in substance use and sobriety among women with concurrent substance use and major depressive disorders as they return to the community from prison.	Relationships Employment Housing Public health and welfare services The criminal justice system
Jones et al., 2021 [47] How do users with comorbidity perceive participation in social services? A qualitative interview study, International Journal of Qualitative Studies on Health & Well-Being	Sweden	Constructivist grounded theory approach was used with semi-structured qualitative interviews	12 participants with concurrent mental health and substance use problems. Nine men and three women, aged 22–65 years. Recruitment through social services, health care providers and user organizations.	To construct a theoretical framework that explains how users with comorbidity of substance use and mental illness/neuropsychiatric disorders portray user participation in social work encounters.	Public health and welfare services
Keesler et al., 2020 [48] “If We Can Feel Like We Have Purpose and We Belong”—Exploring the Experiences of Drug-Involved Individuals in a Rural Jail, Alcoholism Treatment Quarterly	USA	Structured interviews	21 individuals (71% men), incarcerated for drug-related offenses in a rural area. Average 35 years old (SD = 7.13), rural area.	Explore and understand lived experiences prior to incarceration, experiences during incarceration, and outlook for community re-integration.	Belonging Relationships Meaningful activities Public health and welfare services The criminal justice system
Knight et al., 2014 [49] Single room occupancy (SRO) hotels as mental health risk environments among impoverished women: The intersection of policy, drug use, trauma, and urban space, International Journal of Drug Policy	USA	Semi-structured interviews and ethnography	30 women, sub-sample from a larger cohort. Women in the study lived in a range of SRO built environments and had concurrent mental health and substance use problems. Urban area.	Due to the significantly high levels of comorbid substance use and mental health diagnosis among urban poor populations, examining the intersection of drug policy and place requires consideration of the role of housing in drug user mental health.	Housing
Kour et al., 2019 [50] Coping and Negotiating a Sense of Self: Immigrant Men’s Experiences of Living with Co-Occurring Substance Use and Mental Health Disorders in Norway, American Journal of Psychiatric Rehabilitation	Norway	Individual interviews	Ten men with immigrant background and concurrent mental health and substance use problems. Recruited though rehabilitation and treatment centers. Age from 25 to 53 years. Urban area.	This qualitative study aims to explore the lived experiences of being an immigrant and living with concurrent SUDs and MHDs.	Identity Belonging Relationships Employment Public health and welfare services
Kour et al., 2020 [51] Treatment Experiences with Norwegian Health Care among Immigrant Men Living with Co-Occurring Substance Use- and Mental Health Disorders, Substance Abuse: Research and Treatment	Norway	Individual interviews	Ten men with immigrant background and concurrent mental health and substance use problems. Recruited though rehabilitation and treatment centers. Age from 25 to 53 years. Urban area.	This qualitative study aims to explore the treatment experiences of immigrant men living with concurrent SUD and MHD.	Relationships Public health and welfare services
Kozloff et al., 2013 [52] Factors influencing service use among homeless youths with co-occurring disorders, Psychiatric Services	Canada	Focus groups, four.	23 youths with concurrent disorders in urban areas. 20 males, mean age 22.2 (SD 2.1). Recruited through a shelter with short- and long-term facilities where substances were banned on site, an emergency shelter that used a harm-reduction approach, and a drop-in center and health clinic.	Exploring factors influencing service use among homeless youths with concurrent disorders.	Public health and welfare services The criminal justice system
Kronenber et al., 2015 [53] Personal Recovery in Individuals Diagnosed with Substance use Disorder (SUD) and Co-Occurring Attention Deficit/Hyperactivity Disorder (ADHD) or Autism Spectrum Disorder (ASD), Archives of Psychiatric Nursing	Netherlands	Open, in-depth, semi-structured interviews	21 participants were recruited by professionals working at a dual diagnosis, outpatient, treatment facility. Age 27–57, 19 males, two females.	Explore the process of personal recovery in people diagnosed with substance use disorder and comorbid attention deficit/hyperactivity disorder (ADHD) or autism spectrum disorder (ASD).	Relationships Meaningful activities Public health and welfare services
Lawrence-Jones, 2010 [54] Dual diagnosis (drug/alcohol and mental health): Service user experiences, Practice.	England	Individual topic-focused interviews	Six service users recruited through a statutory, multi-disciplinary substance use agency. Two females, four males, ages ranged from 37 to 61. concurrent mental health and substance use problems.	Develop insight into the lived experience of dual diagnosis by exploring the narratives of service users. Difficulties in accessing services in the context of the noted tendency for service users to be shunted between services; experiences of seeking support for both issues within services that typically have a more narrowly defined remit; and experiences of stigma.	Public health and welfare services
Luciano et al., 2014 [55] Long-term Sobriety Strategies for Men with Co-occurring Disorders, Journal of Dual Diagnosis	USA	semi-structured interviews	12 men with cooccurring psychosis and substance use disorder who achieved and maintained sobriety for at least one year, participating in residential or outpatient treatment at a private, non-profit integrated treatment clinic. Age 23 to 42 years.	Explore strategies for relapse prevention as described by men with concurrent disorders who achieved one or more years of sobriety.	Relationships
Luciano & Carpenter-Song, 2014 [56] A qualitative study of career exploration among young adult men with psychosis and co-occurring substance use disorder, Journal of Dual Diagnosis	USA	In-depth interviews	Twelve young adult men aged 18 to 35 years, mean age of 26 (SD = 3) with concurrent disorders. Recruited from an integrated treatment center.	Explore the meaning and importance of career exploration and career development in the context of integrated treatment for young adults with early psychosis and substance use disorders (i.e., concurrent disorders).	Identity Belonging Employment
Milani et al., 2020 [57] A qualitative longitudinal study of the first UK Dual Diagnosis Anonymous (DDA), an integrated peer-support programme for concurrent disorders, Advances in Dual Diagnosis	England	Qualitative interviews, part of a mixed methods evaluation	Six DDA members, two females, four males. Urban area.	Explore the impact and mechanism of change of the program through the perspective of DDA attendees, facilitators, and the funding commissioners.	Identity Belonging Relationships Meaningful activities Employment Housing
Ness et al., 2017 [58] “Sorting things out together:” Young Adults’ Experiences of Collaborative Practices in Mental Health and Substance Use Care, American Journal of Psychiatric Rehabilitation	Norway	Individual qualitative in-depth interviews	Seven young adult service users, two females and five males, aged between 20–30 with concurrent mental health and substance use problems. Recruited from the Mental Health, Substance Use and Child and Family Services	To provide insights regarding what young adult service users who receive mental health and substance use services find helpful, and factors that facilitate, as well as factors that disrupt, treatment engagement.	Public health and welfare services
Nordaunet & Sælør, 2018 [59] How meaningful activities influence the recovery process, Advances in Dual Diagnosis	Norway	Phenomenological-hermeneutic-style in-depth interviews	Five men with concurrent conditions living a residential care facility. Ages ranged from early forties to mid-60s. Semi-urban area.	The purpose of this paper is to explore two research questions: how do people with concurrent substance abuse and mental health disorders (concurrent conditions) experience and describe meaningful activities? And how do meaningful activities influence the recovery process?	Identity Belonging Relationships Meaningful activities Public health and welfare services The criminal justice system
O’Sullivan et al., 2013 [60] Lived experiences of recalled mentally disordered offenders with dual diagnosis: A qualitative phenomenological study, The Journal of Forensic Psychiatry & Psychology	England	Semi-structured interviews	Five males, ages 26–42, offenders with dual diagnosis. Recalled service users from a medium secure unit. Urban area.	Explore the lived experience of mentally disordered offenders with dual diagnosis.	Identity Belonging Public health and welfare services
Ogundipe et al., 2020a [61] Recovery on the Pitch: Street Football as a Means of Social Inclusion, Journal of Psychosocial Rehabilitation and Mental Health	Norway	Focus groups	51 persons experiencing mental health and/or substance abuse challenges who played in four street football teams. 48 males, 3 females, most between 40–50 years. semi-urban/rural area.	What experiences do players have with participation in street football teams in relation to their recovery processes?	Identity Belonging Relationships Meaningful activities Public health and welfare services
Ogundipe et al., 2020b [62] "Come together": a thematic analysis of experiences with belonging, Advances in Dual Diagnosis	Norway	Individual semi-structured interviews with target group, focus group with professionals not extracted.	Five persons with concurrent mental health and substance abuse problems living in supportive housing in a Norwegian district. Three females, two males.	Explore, describe and interpret two research questions: How do persons with concurrent g mental health and substance abuse problems, living in supportive housing, experience belonging? How do residential support staff experience promoting a sense of belonging for persons with concurrent mental health and substance abuse problems, living in supportive housing?	Identity Belonging Meaningful activities Economy Housing Public health and welfare services
Padgett et al., 2008 [63] Social relationships among persons who have experienced serious mental illness, substance abuse, and homelessness: Implications for recovery, American Journal of Orthopsychiatry	USA	Within- and across-case analyses of longitudinal data from qualitative interviews. Three in-depth interviews at 0, 6 and 12 months.	41 dually diagnosed individuals entering four residential programs to exit homelessness and receive needed services. 29 males, 12 females, mean age 41, with a range of 21–60. Urban area.	Investigating the role of positive social relationships in recovery among homeless individuals with serious mental illness and comorbid substance abuse.	Identity Relationships Employment Economy Public health and welfare services
Padgett et al., 2016 [64] Complex Recovery: Understanding the Lives of Formerly Homeless Adults with Complex Needs, Journal of Social Distress & the Homeless	USA	Burawoy’s extended case method conducted on in-depth interviews	74 formerly homeless adults living in housing programs in an urban area.	Examine mental health recovery in formerly homeless adults with serious mental illness and concurrent substance abuse.	Identity Relationships Housing Public health and welfare services The criminal justice system
Patterson et al., 2015 [65] Exiting homelessness: Perceived changes, barriers, and facilitators among formerly homeless adults with mental disorders, Psychiatric Rehabilitation Journal	Canada.	Semi-structured interviews. Interviewed within 1 month of enrollment and again 18 months later. Recruited through referral from a variety of agencies that serve homeless adults	43 participants 25 men (58%), 16 women (37%), and two (5%) transgendered individuals. 28 in Housing First, 15 TAU. Age ranged from 21 to 66 years (M = 43 years). Urban area.	This study examines key themes from narrative interviews conducted with 43 homeless adults in Housing First with intensive support or treatment as usual (no housing or support through the study). Two main questions: (a) What changes (if any) did participants perceive over 18 months post randomization? (b) What factors facilitated or hindered change?	Identity Relationships Housing Public health and welfare services
Semb et al., 2019 [66] Communal invalidation of young adults with co-occurring substance abuse and mental health issues, Disability & Society	Norway	Semi-structured interview, two interviews. Recruited through the municipal health and social services.	Two participants, age 21 and 23, with concurrent mental health and substance use problems. Urban area.	The article explores how young adults with concurrent substance use and mental health problems experience and describe their own and others’ contributions to their sense of community belonging.	Identity Belonging
Semb et al., 2021 [67] Resist or Adapt? A Narrative Analysis of Endeavors for Belonging Among Young Adults with Co-Occurring Substance Use and Mental Health Problems, Journal of Psychosocial Rehabilitation and Mental Health	Norway	In-depth interviews, six interviewed twice. Recruited through the municipal health and social services.	Seven participants with concurrent mental health and substance use problems. Age 18–30 years, median 22. Urban area.	This study explores how young adults with co-concurrent substance abuse and mental health issues experience the challenges of belonging to their local communities.	Belonging Employment
Shelton, 2004 [68] Experiences of detained young offenders in need of mental health care, Journal of Nursing Scholarship	USA	Focus groups, six groups, 12 interviews	30 young people who were receiving mental health services through a juvenile justice system. 10 female, 20 male, between the ages of 13 and 17 years. The majority of them have concurrent mental health and substance use problems.	To explore the experiences of young people detained in the juvenile justice system and in need of mental health services.	Identity Public health and welfare services The criminal justice system
Skogens et al, 2018 [69] Initiating and maintaining a recovery process–experiences of persons with dual diagnosis, Advances in Dual Diagnosis	Sweden	Semi-structured individual interviews.	40 people with concurrent mental health and substance use problems having experienced positive change. 13 women and 27 men between 26 and 62 years old (m = 43). Urban area. Recruited through in-patient and outpatient units in psychiatric care and AOD treatment.	Investigate the internal and social factors that persons with experience from severe mental illness and alcohol and other drugs problems, and who have received treatment for these problems, describe as important for initiating and maintaining a recovery process.	Identity Relationships Meaningful activities Economy Housing The criminal justice system
Staiger et al., 2011 [70] Improving services for individuals with a dual diagnosis: A qualitative study reporting on the views of service users, Addiction Research & Theory	Australia	Semi-structured interviews	Sub-sample of larger study, 44 participants with concurrent mental health and substance use problems. 21 females (23–49 years, M = 34,22 SD = 7.88), 23 males (26–55 years, M = 38.17, SD = 7.57).	Explore service experiences (barriers to treatment and suggestions for improvements).	Relationships Public health and welfare services
Stenius et al., 2005 [71] Social roles in women’s lives: changing conceptions of self, Journal of Behavioral Health Services & Research	USA	Structured interviews. 105 women in integrated site, 120 women in comparison site, lower numbers at follow up.	Women enrolled in the study all had mental health and substance use diagnoses, a history of physical or sexual abuse, and had received services in the mental health or substance abuse treatment system. Age range 21–61, M = 37.	As part of an effort to improve services and outcomes for women with histories of trauma and concurrent mental health and substance abuse disorders, the Franklin County Women and Violence Project sought to assess women’s perceptions about their social roles and provide them with opportunities to adopt valued social roles.	Identity Relationships Meaningful activities Employment
Strickler et al., 2009 [72] First person accounts of long-term employment activity among people with dual diagnosis, Psychiatric Rehabilitation Journal	USA	Self-reports at yearly interviews occurring over a 16-year period. In-depth interview occurred at 16 years.	120 people with severe mental illness and concurrent substance use disorder recruited from community mental health clinics. Rural and urban regions.	The purpose of this study is to elicit and examine first person accounts of work activity over a 16-year period from people with dual diagnosis, who were not selected for employment readiness or vocational interests	Identity Belonging Employment
Sælør & Skatvedt, 2019 [73] Thresholds of hope: stories of lacking generosity, Social Work in Mental Health	Norway	In-depth interviews	Eight men and one woman, all with concurrent mental health and substance use problems. Ages ranging from their early 20s to approximately 60, were ultimately recruited as participants. recruited in collaboration with practitioners and leaders within municipal mental health and substance use service.	Explore how service users experience barriers to help and assistance, and to determine the manner by which these barriers may influence their experiences of hope.	Identity Belonging Public health and welfare services
Sælør et al., 2021 [74] A tale from the Glass Dome: A narrative analysis of social housing, living conditions and recovery, Nordic Welfare Research	Norway	Individual semi-structured interviews interviewed twice.	Eight persons with concurrent mental health and substance use problems received services from a community outreach team. Age ranged from the early twenties to late fifties, women and men.	This article explores how housing circumstances in Norway may influence recovery for persons experiencing concurrent mental health and substance abuse problems.	Identity Relationships Economy Housing Public health and welfare services
Topor et al., 2021 [75] Micro-affirmations and recovery for persons with mental health and alcohol and drug problems: User and professional experience-based practice and knowledge, International Journal of Mental Health and Addiction	Sweden	Individual interviews	13 women and 27 men aged between 26 and 62 with concurrent mental health and substance use problems. Recruited through social and psychiatric in- and outpatient services. The participants had to have experienced a positive change concerning their problems and this change had to be confirmed by a professional. Urban region	Explore the concrete construction of professional helpful relationships, and how they are developed in daily practice.	Identity Relationships Economy Public health and welfare services
Tsai et al., 2012 [76] Housing preferences and choices among adults with mental illness and substance use disorders: a qualitative study, Community Mental Health Journal	USA	Semi-structured interviews with 20 from supervised and 20 from independent housing	40 adults with dual disorders, living in either supervised or independent housing, with a history of homelessness. Urban area. Most black males with mean age 46.3 years (SD = 7.7) and mean residential tenure of 33.2 months (SD = 31.4) in their current housing.	Examine housing preferences, decision making processes surrounding housing choices, and perceived barriers to housing.	Housing
VanDeMark, 2007 [77] Policy on reintegration of women with histories of substance abuse: a mixed methods study of predictors of relapse and facilitators of recovery, Substance Abuse Treatment, Prevention, & Policy	USA	Mixed methods, data extracted from qualitative components of structured interviews.	325 women with histories of substance abuse, majority with concurrent mental health problems.	Examine the predictors of relapse and the facilitators of recovery.	Identity Relationships Employment Economy Public health and welfare services The criminal justice system
Vandevelde, 2010 [78] Villa Voortman: Carte blanche or not? Therapeutic Communities	Belgium	Visitors and professionals interviewed; data extracted from video-recorded interviews with visitors	19 people, four persons females, 15 males, between 20 and 60 years old. All with concurrent mental health and substance use problems. Urban area.	The purpose of this paper is to investigate the position of Villa Voortman in the treatment continuum for dually diagnosed clients. Two research questions are addressed: how does Villa Voortman operate? And how do clients perceive the Villa?	Belonging Relationships Public health and welfare services
Villena & Chesla, 2010 [79] Challenges & struggles: lived experiences of individuals with mental illness, substance abuse, and general medical conditions, Archives of Psychiatric Nursing	USA	Individual interviews	20 people, 11 men (55%) and 9 women (45%), with a mean age of 51 years, with concurrent mental health and substance use problems, and medical conditions. Recruited from community treatment centers and supportive housing sites.	Understand, describe, and illustrate the social and structural barriers that individuals with COD of mental illness, substance abuse, and general medical conditions encounter regarding their health care.	Housing Public health and welfare services
Von Greiff et al., 2020 [80] Supporting recovery in social work with persons having co-occurring problems–clients’ and professionals’ perceptions, Nordic Social Work Research	Sweden	Semi-structured individual interviews with clients and professionals, data extracted from clients’ interviews.	40 people with concurrent mental health and substance use problems having experienced positive change. 13 women and 27 men between 26 and 62 years old (m = 43). Urban area. Recruited through in-patient and outpatient units in psychiatric care and AOD treatment.	Explore how clients with concurrent problems describe the importance of treatment factors for the recovery process and how these descriptions relate to professional descriptions.	Relationships Public health and welfare services

### Stage 5 –Collating and summarizing the results

SNB employed an explorative and inductive content analysis approach including the stages of preparation, organizing and reporting [23], following recommendations for analysis in qualitative scoping reviews [24]. After collecting and reading through the data, the material was coded and categorized both by facilitators and barriers, before identifying overarching themes and subthemes, and interpreting these. All authors were consulted in this process. The software F4analyse [25] was used for analysis. Results are shown in Table 2, followed by narrative descriptions in the section below, in line with recommendations for reporting in scoping reviews [24].

## Results

The results concern barriers to and facilitators of social inclusion as experienced by people with concurrent mental health and substance use problems. Our analysis yielded three main themes: (1) The intrapersonal baseline, (2) Components of social inclusion, and (3) Systemic failure or success, with subthemes for each (see Table 1 for main themes and subthemes, and Table 2 for included articles and results).

### The intrapersonal baseline

This theme highlights the importance of including processes rooted in the individual when making efforts to promote social inclusion, as related to two subthemes: (1) Identity, and (2) Belonging.

#### Identity

Identity affects social inclusion via self-perception of being someone who is, or is not, socially capable, of value, or desirable to others. Participants in the included studies often perceived themselves as worthless and unwanted. This was often due to loss of social roles, connected with a sense of hopelessness or a wish “to be normal” or “fit in”. They experienced this as creating a distance between themselves and others.

A negative identity was described as a barrier to social inclusion via its effect on behavior, with others’ reactions to out-of-norm behavior confirming self-perception as an unwanted and ‘second-class citizen’. Trying to change or control this behavior was by some experienced as challenging, lacking knowledge or resources to adjust it. These negative reactions came not only from people in the mainstream community, but from service providers as well. Some perceived treatment or hospitalization as confirming their “disturbed identity,” being someone who is not normal. Receiving kindness could be confusing for some if it went against their established identity of being unworthy.

To facilitate social inclusion through redefining self-perception, some pointed to the importance of skill building to increase a sense of self-efficacy. Others spoke of the importance of having socially valuable roles through working or engaging in meaningful activities, and having satisfactory housing. Some said receiving flexible help and being treated with dignity through person-oriented services helped them redefine themselves more positively and counteract the sense of being a burden to society. They also spoke of establishing new, healthy identities that promote their social inclusion through gaining greater knowledge of mental health and substance use problems, practicing self-forgiveness, being recipients of acts of kindness, offering it to others, and internalizing these experiences, thus creating identities outside of illness.

#### Belonging

A sense of belonging supports social inclusion, as evident in the material. Many people reported a lifelong sense of not belonging, related to negative experiences in their families, school, or community giving them conflicting feelings of both wanting and not wanting to belong in mainstream community. Stigma and racism, often experienced as worse in rural areas, were described as barriers to belonging. Other barriers were homelessness, housing, lack of an educational degree, family connections, network, or jobs, and having lived a different life from others, making it difficult to connect. Some preferred finding a sense of belonging among other outsiders who also had problems and saw themselves as having limited possibilities to connect with people outside of this. Some spoke of a desire to start over, being given a clean slate from society, avoiding the stigma and the barrier that come with it.

People spoke of achieving a sense of belonging and inclusion through participating in meaningful activities, therapeutic groups, or self-help groups, where they shared common interests, challenges, and experiences. Some, however, wondered if participation in arranged activities and charities with peers functioned as a barrier to belonging into the mainstream community. Others spoke of working or receiving support from family and friends as a facilitator. A few emphasized collective community efforts done for their benefit to help them achieve a sense of belonging, pointing to the role of other people in the community, and the importance of acting on one’s healthy values and being met with them by others.

### Components of social inclusion

This theme involves components of social inclusion, with the subthemes of (1) Relationships, (2) Meaningful activities, (3) Employment, and (4) Economy. These components are crucial for social inclusion as they address both subjective and objective aspects.

#### Relationships

For many of the participants in the included studies, challenging relationships with family or partners, or multiple relational traumas through losing contact with family, partners, friends, and children or due to deaths, created a lasting sense of disconnectedness and a barrier to social inclusion. Many found it difficult to establish new relationships or re-connect with people due to bad experiences, including with service providers. This was associated with lack of support, confidence, or knowledge as to how to go about it, leading to greater isolation and exclusion. Others avoided starting relationships out of preference, fear of relapse, self-protection in hostile environments, estrangement, or fear of rejection. People also found building relationships difficult due to their mental health or substance use related problems, feeling they had nothing to offer.

Some found it easier to establish healthy relationships with peers though participating in activities or peer groups supported by a mutual understanding and non-judgmental acceptance. These relationships helped build confidence and social skills and were starting points for connecting with others. Ending unhealthy relationships, overcoming a lack of trust in others and experiences with not being trusted was necessary to facilitate connection. For some, reconnection with family depended on creating a sphere in which struggles could be addressed and understood by family members. Involving family members and other significant people in treatment could further support social inclusion.

#### Meaningful activities

Engaging in meaningful activities was often described as a starting point for promoting social inclusion. Through participating in different activities, especially with peer support, people experienced increased self-confidence, social skills, coping mechanisms for illness, and motivation to participate in other activities such as voluntary work. This functioned as a gateway to employment for some. In addition, it could create a sense of normalcy, better quality of life and belonging via offering access to valuable social roles and an expanded social network.

Barriers to social participation were associated with insecurities about social skills, lack of money, transportation problems, and limited choice of activities, the last especially for people in rural areas. Feeling forced to participate in activities that one was not interested in could be a barrier to participating in other arranged activities. Related to this, focusing on activities themselves rather than the social aspects of them helped some people overcome social insecurities and engage. Receiving information and advice about relevant activities and motivational support through professionals, peer-staff and peers also facilitated participation and social inclusion.

#### Employment

Employment supported achievement of self-respect, restoration of relationships with family, provision for children, being a good role model, and contributing to society. Most of the barriers and facilitators mentioned in relation to participating in meaningful activities also applied to employment, such as insecurities about social and practical skills.

Other barriers mentioned were stigma, racism, side effects of medications, poor physical health, criminal records, and lack of social and familial support. Some people spoke of not finding satisfactory work because of not having kept up with changes in the workplace, while others feared losing benefits and not earning enough to cover their loss. Others avoided work because of a lack of perceived ability, lack of hope, finding increased contact with others too stressful, or wanting to focus on other activities, family, or children.

People spoke of paid work, not training programs, along with support to maintain financial safety, as supporting their self-confidence. Finding the ‘right job,’ receiving positive support privately and from professionals, and gaining a distance from substance use, was improving their work performance and facilitated social inclusion.

#### Economy

Economic status is important to promote social inclusion and prevent social exclusion seen as poverty and lacking financial security pushed many toward homelessness and the underground economy. This worsened their marginalization and lack of engagement with their community. Benefits were the main income for many, but these could be hard to get and easy to lose. For women especially, financial solvency affected their opportunities to provide for their children, an important factor in staying connected to society.

Doing better economically facilitated social inclusion, helping people choose safe places to live. Having money helped people engage with others, participate in social gatherings and establish reciprocal relationships by making it possible for them to contribute to them. Having money also supported people’s improved quality of life and ability to maintain their health through stabilizing their living situations.

### System failure or success

Systems of care could support or inhibit people’s social inclusion through the sub-themes of (1) housing, (2) public health and welfare services, and (3) the criminal justice system.

#### Housing

Housing, along with autonomy, a sense of dignity, safety, and stability, undercut people’s feeling of living on the outskirts of society and promote social inclusion. Some preferred living in supervised housing, others wanted independent housing. Some preferred to live among the general population, others among peers.

Supervised housing may impede social inclusion if not adapted to the person’s needs. Some preferred homelessness to unsafe, undignified housing. Others self-isolated in supportive housing due to stigmatizing encounters with staff or easy access to substances challenging their recovery. On the other hand, some experienced a sense of community in supported housing, with peer and staff support. This was often enhanced by collaboration between mental health and substance use service providers and housing staff, and enforcement of housing rules supporting positive social interaction. When housed in a functioning program, people often found increased hope for the future, and this sometimes led to reconnection with family, returning to school, and engagement in meaningful activities including voluntary work or paid work.

Some people spoke of procuring independent apartments after tackling barriers such as financial issues, rejection of housing vouchers, criminal history, and stigma. Maintaining independent housing could be overwhelming, too, and tempt people to return to homelessness. Experiencing mental health problems when stably housed, sometimes influenced by reduced substance use, point to the importance of continued professional support.

#### Public health and welfare services

People cited problems in their contacts with public health and welfare services as a barrier to social inclusion. Problems included difficulties gaining access to mental health and substance use services and lack of integration of care for those who needed both. Some felt disempowered after negative encounters with service providers across years of seeking services, leading them to distrust the system and avoid engaging in treatment. When seeking treatment, bureaucratic delays and challenges, and issues with health insurance and finances were other reported barriers, the latter more pronounced in non-Nordic countries. Difficulties gaining access to treatment, a lack of person-centered care and limited involvement in decision-making, the perceived dominance of the medical model, coercion, and lack of expertise with treating concurrent conditions, often led people to end their treatment prematurely. In addition, many reported feeling stigmatized and mistrusted by staff due to their previous histories, or experienced low expectations from staff regarding their potential to contribute to society.

People also spoke of facilitating experiences. Receiving integrated and person-centered care, they said, supported their engagement in treatment. They cited being involved, experiencing collaboration and support in meetings with service providers across agencies, focusing on resources, personal skills, and on emotional and practical coping skills relevant to daily life as especially helpful. Staff whom they saw as being trusting and trustworthy, supportive, and respectful facilitated recovery and social inclusion, in addition to them having a sense of humor, patience and a non-judgmental attitude. Peer support, vocational and recreational activities, and outreach work also helped some gain access and stay connected to services, and could be steppingstones to connection with others. Having staff acknowledge their alienation from society helped them become engaged in services, some said, and supported their efforts to achieve social inclusion.

#### The criminal justice system

Some people reported experiences with the criminal justice system, and for some of these, court requirements to seek treatment or prison terms supported their rehabilitation and social reintegration. Others, though, said the legal system can’t provide treatment and that substance use should be decriminalized to help the overall chances of living a decent life. Some simply did not have faith in the criminal justice system, leaving them feeling disempowered with little hope of social reintegration.

People offered few facilitating factors. Some spoke of support from treatment programs while others spoke of familial support. Non-judgmental support from staff was helpful, albeit mostly compared to the draconian practice of isolation. For women, same-gender support was important, both from people not using substances and from peers who were. Some spoke of being able to establish a supporting network while imprisoned and re-connecting with supportive people.

People said continued substance use or relapse following release were barriers to social reintegration. Rehabilitation in jail, they said, was difficult due to easy access to substances and lack of treatment. These factors were worsened by experiences of stigma and being poorly treated by staff and the system. Longer prison terms also created a barrier because of estrangement in family relationships.

People spoke of insufficient support from public services for transition back to their communities, including a lack of emotional and practical support for securing financing, housing, and work. The mainstream community, they said, refused to give them a second chance, thus limiting contact with people outside their old networks.

## Discussion

Our study of first-person experiences among people with concurrent mental health and substance use problems guides us in the implications and recommendations we discuss in this section. Most of the included studies were conducted in the U.S., Great Britain, and Nordic countries, suggesting caution regarding their relevance to other settings. Why research is more prevalent in these countries compared to others raises questions as to how social inclusion is experienced and addressed in other countries, pointing to the need for further investigation. Additionally, most studies did not investigate potential differences in subsamples by factors such as gender, age, or context. Therefore, we cannot differentiate among subgroups, in turn challenging their implications for practice. Further, social inclusion was largely studied indirectly as part of general recovery processes, revealing a lack of holistic investigation of social inclusion. Finally, many studies were conducted within treatment or housing programs, therefore it should come as no surprise that our results point to experiences with health and welfare systems as a main barrier or facilitator of social inclusion.

The results show barriers on all levels from the individual to the societal, with barriers often clearer than facilitators. Participants often had lifelong experiences with social exclusion, often related to people’s mental health and substance use problems, lack of resources, and worldviews stemming from a life at the margins of society. These were often accompanied by relational trauma, stigma, and lack of trust that followed the persons in meetings with service providers, who sometimes validated people’s lack of trust in others and their internalized stigma. Societal barriers included limited access to and over-dependence on services, while access to services and dignified treatment while receiving them were facilitators of inclusion. However, people saw support from peers or peer staff as equally, or more important than support from professionals.

### Implications for research

Our finding points to the importance of further exploration of how factors such as age, gender, type of problems, generational and sociodemographic characteristics influence social inclusion. Such findings could promote person- and context oriented and socially acceptable services. Additionally, future research should investigate the overarching societal structures in addition to systemic, supporting efforts to create practical solutions. Action research including participation of people with lived experience and aimed at improving support and service provision may help to close the gap between research and practice.

### Implications for practice

It is striking that so much knowledge can be gained through talking to people who are socially excluded, and that there is so little capacity, at present, to implement this knowledge. There is an urgent call for systemic change and more robust implementation of socially oriented, knowledge-based treatment models. This is important considering people’s negative experiences with the current fragmented services and system of care, which undermined their access to social inclusion and participation in the society. We need to transform current systems of care, but we also need to support people’s transition to participation in mainstream society, with the critical involvement of people with lived experience in these efforts. The Citizenship framework [81, 82] is receiving attention as a way of addressing exclusion for marginalized groups, e.g. in Scotland [83], Spain [84] Norway [32, 85], and the U.S [81, 82], and serves as a good example of practice.

### Implications for policies

Our findings seem to indicate violations and lack of enforcement of human rights in relation to social exclusion. There is a need to develop policies that secure fundamental human rights and promote mental health and social inclusion through improving living conditions such as economy, housing, and treatment, and ensuring that legal policies do good, not harm. Societal structures and attitudes build upon the creation of parallel communities, excluding the target group from mainstream society. Parallel overarching societal processes may be significant points of interest in addressing policies for social inclusion for this target group, and others. These processes include deinstitutionalization, the construction of fragmented and often inaccessible health and welfare systems, increasing inequities in health, economy and social welfare, and the current financial political climate including the promotion of individualistically oriented culture. Such processes negatively affect societal attitudes and sense of responsibility towards those less fortunate and influence the target group’s access to mainstream society. Such processes and statuses, combined with excluded persons learned mistrust in services which, to further complicate matters, are subject to cutbacks in financial challenging times, are a cause for worry.

The role of civic society in promoting social inclusion is rarely explored but should be. Possible starting points to address these issues are Prilleltensky´s [86] proposals for a shift from a “me-culture” to a “we-culture” to promote mattering, wellbeing, and connectedness, the work of Davidson [87] pointing to the importance of “simply being let in” as opposed to “fitting in”, and Prilleltensky et al.’s [88] work on the conditions (distributive, procedural and corrective justice) to promote mattering and well-being. Given the barriers related to our main themes, as seen in association with effects of “me-cultures”, it would be interesting to explore how civil society could facilitate social inclusion for socially excluded people with complex problems. Such explorations should and must involve individuals with lived experience.

### Strengths and limitations

Our study has some methodological limitations. In general, the different steps and processes that result in a scoping review are not linear. In conducting a scoping review, a sensitive search is of great importance. Our search strategy is a combination of a sensitive search, and a more specific search performed some weeks apart. We consider this to be a strength of our review, as it incorporates the iterative nature of searching for relevant literature. We have not hand searched key journals as suggested in the framework but consider our research question to be of an interdisciplinary character, and as such identification of key journals is difficult. This fact is also reflected in the large number of unique journals included in the review. The analyses showed high levels of common experiences expressed across different study populations, pointing to high internal validity in the study. Another strength is our team-based approach with complementary perspectives, including the peer researcher’s, enhancing the quality of review process. However, there are some limitations on generalizability, as most of the studies were conducted in the USA, UK, and Nordic countries, mostly seen as high-income countries. In addition, few studies addressed potential differences in subsamples by factors such as gender, age, or context, demanding precautions.

## Conclusion

Promoting social inclusion for persons with mental health and substance use problems is a complex matter demanding multidisciplinary approaches and actions. Massive research, practice and policy efforts must be undertaken to address social inclusion both directly and in a broad social context, aimed at breaking down barriers and building facilitators. More effort to include the social context in individual treatment and follow-up-care is needed as well. We challenge researchers, practitioners, and policy makers to take further steps in addressing, implementing knowledge, and handling the matter of social inclusion among people with concurrent mental health and substance use problems, as it is not only a societal concern but also a fundamental human right.

## Supporting information

S1 Checklist Preferred Reporting Items for Systematic reviews and Meta-Analyses extension for Scoping Reviews (PRISMA-ScR) checklist. (PDF)

S1 File Search strategies. (PDF)
