# Supplementary material for: Barriers and facilitators for social inclusion among people with concurrent mental health and substance use problems. A qualitative scoping review
Source: PLoS One. 2024 Dec 16;19(12):e0315758. doi: 10.1371/journal.pone.0315758 (PMC11649107; doi:10.1371/journal.pone.0315758)
Supplement: S1 File — (PDF) [file pone.0315758.s002.pdf]

No man is an island: Barriers and facilitators for social inclusion among people with concurrent mental health and substance use problems: A scoping review of qualitative research

Broad search conducted February/March 2023

SCOPUS:

TITLE-ABS-KEY ("Dual diagnosis" OR coccur* OR co-occur* OR comorbid* OR coexist* OR concurr*) AND TITLE-ABS-KEY ("mental disorder*" OR "mental illness*" OR "mental health" OR "psychiatric illness*" OR "psychiatric disorder*") AND TITLE-ABS-KEY ("substance use disorder*" OR "substance use" OR "substance abuse" OR "substance misuse" OR "alcohol use" OR "drug use" OR "substance use" OR "alcohol use" OR "alcohol misuse" OR "alcohol abuse") AND TITLE-ABS-KEY (inclusion OR "social accept*" OR participation OR "social participat*" OR "recovery" OR "community integration" OR stigma* OR belong* OR "social ident*") AND TITLE-ABS-KEY (exclusion OR "social exclusion" OR isolat* OR "social isolat*" OR "social marginal*" OR marginal* OR ostrac* OR "social withdraw*" OR "social depriva*")

EMBASE

Embase <1980 to 2023 Week 12>

1	"Diagnosis, Dual (Psychiatry)"/	15
2	co-occur*.mp.	47963
3	cooccur*.mp.	2583
4	exp Comorbidity/	377737
5	comorbid*.mp.	557266
6	concurrent.mp.	203947
7	coexist*.mp.	110687
8	1 or 2 or 3 or 4 or 5 or 6 or 7	890697
9	exp mental disorders/	2514694
10	"mental illness*".mp.	52509
11	exp Mental Health/	222999
12	"psychiatric illness*".mp.	14864
13	"psychiatric disorder*".mp.	74162
14	9 or 10 or 11 or 12 or 13	2635893
15	exp "substance use disorder"/	256314
16	((substance or drug or alcohol) adj2 (abuse or misuse)).mp.	197219
17	"alcohol use".mp.	68355
18	"drug use".mp.	194862
19	"substance use".mp.	67861
20	15 or 16 or 17 or 18 or 19	596443
21	exp social acceptance/	4187
22	("social inclusion" or "recovery" or "social recovery" or citizenship).mp.	727425
23	"social participation".mp.	11917
24	exp social deprivation/	30763
25	exp social exclusion/	2731
26	"social ident*".mp.	3104
27	belong*.mp.	304063
28	stigma*.mp.	74238

29	21 or 22 or 23 or 24 or 25 or 26 or 27 or 28	1144319
30	8 and 14 and 20 and 29	2500

PSYCINFO

APA PsycInfo <1806 to March Week 3 2023>

1	"Diagnosis, Dual (Psychiatry)"/	0
2	co-occur*.mp.	19534
3	cooccur*.mp.	593
4	exp Comorbidity/	39093
5	comorbid*.mp.	91461
6	concurrent.mp.	39762
7	coexist*.mp.	9927
8	1 or 2 or 3 or 4 or 5 or 6 or 7	150599
9	exp mental disorders/	1014403
10	"mental illness*".mp.	54023
11	exp Mental Health/	86590
12	"psychiatric illness*".mp.	9171
13	"psychiatric disorder*".mp.	43947
14	9 or 10 or 11 or 12 or 13	1097726
15	exp "substance use disorder"/	121268
16	((substance or drug or alcohol) adj2 (abuse or misuse)).mp.	107791
17	"alcohol use".mp.	58931
18	"drug use".mp.	38423
19	"substance use".mp.	73766
20	15 or 16 or 17 or 18 or 19	224047
21	exp social acceptance/	10964
22	("social inclusion" or "recovery" or citizenship).mp.	98648
23	"social participation".mp.	4071
24	exp social deprivation/	10442
25	exp social exclusion/	2184
26	14 or 20	1167960
27	stigma*.mp.	43104
28	belong*.mp.	39065
29	"social ident*".mp.	23836
30	21 or 22 or 23 or 24 or 25 or 27 or 28 or 29	216303
31	8 and 14 and 20 and 30	1474

MEDLINE
Ovid MEDLINE(R) ALL <1946 to March 29, 2023>

1	"Diagnosis, Dual (Psychiatry)"/	3728
2	co-occur*.mp.	38718
3	cooccur*.mp.	1384
4	exp Comorbidity/	126387
5	comorbid*.mp.	297583
6	concurrent.mp.	139822
7	coexist*.mp.	93799
8	1 or 2 or 3 or 4 or 5 or 6 or 7	555860
9	exp mental disorders/	1418853
10	"mental illness*".mp.	39988

11	exp Mental Health/	59394
12	"psychiatric illness*".mp.	10157
13	"psychiatric disorder*".mp.	50779
14	9 or 10 or 11 or 12 or 13	1490850
15	exp "substance use disorder"/	308361
16	((substance or drug or alcohol) adj2 (abuse or misuse)).mp.	100105
17	"alcohol use".mp.	45217
18	"drug use".mp.	53447
19	"substance use".mp.	49454
20	15 or 16 or 17 or 18 or 19	408029
21	exp social acceptance/	194
22	("social inclusion" or "citizenship" or "recovery").mp.	584195
23	"social participation".mp.	6520
24	exp social deprivation/	3240
25	exp social exclusion/	25217
26	21 or 22 or 23 or 24 or 25	614754
27	8 and 14 and 20 and 26	974

Print Search History CINAHL with Full Text

MY

EBSCOhost

Wednesday, August 16, 2023 6:50:17 AM

#	Query	Limiters/Expanders	Last Run Via	Results
S44	S42 AND S43	Expanders - Apply equivalent subjects Search modes - Find all my search terms	Interface - EBSCOhost Research Databases Search Screen - Advanced Search Database - CINAHL with Full Text	220
S43	qualitative or interview* or "focus group*" or "first person" or experienc* or narrative*	Expanders - Apply equivalent subjects Search modes - Find all my search terms	Interface - EBSCOhost Research Databases Search Screen - Advanced Search Database - CINAHL with Full Text	953,096
S42	(S27 OR S28 OR S29 OR S30 OR S31 OR S32 OR S33 OR S34 OR S35 OR S36 OR S37) AND (S9 AND S38 AND S39 AND S40)	Limiters - Published Date: 20000101-20231231 Expanders - Apply equivalent subjects Search modes - Find all my search terms	Interface - EBSCOhost Research Databases Search Screen - Advanced Search Database - CINAHL with Full Text	429
S41	(S27 OR S28 OR S29 OR S30 OR S31 OR S32 OR S33 OR S34 OR S35 OR S36 OR S37) AND (S9 AND S38 AND S39 AND S40)	Expanders - Apply equivalent subjects Search modes - Find all my search terms	Interface - EBSCOhost Research Databases Search Screen - Advanced Search Database - CINAHL with Full Text	443
S40	S27 OR S28 OR S29 OR S30 OR S31 OR S32 OR S33 OR S34 OR S35 OR S36 OR S37	Expanders - Apply equivalent subjects Search modes - Find all my search terms	Interface - EBSCOhost Research Databases Search Screen - Advanced Search	Display

			Database - CINAHL with Full Text	
S39	S18 OR S19 OR S20 OR S21 OR S22 OR S23 OR S24 OR S25 OR S26	Expanders - Apply equivalent subjects<br>Search modes - Find all my search terms	Interface - EBSCOhost Research Databases<br>Search Screen - Advanced Search<br>Database - CINAHL with Full Text	Display
S38	S11 OR S12 OR S13 OR S15 OR S16	Expanders - Apply equivalent subjects<br>Search modes - Find all my search terms	Interface - EBSCOhost Research Databases<br>Search Screen - Advanced Search<br>Database - CINAHL with Full Text	Display
S37	"belong*" OR "recovery"	Expanders - Apply equivalent subjects<br>Search modes - Find all my search terms	Interface - EBSCOhost Research Databases<br>Search Screen - Advanced Search<br>Database - CINAHL with Full Text	Display
S36	"social identi*" OR (MH "Social Identity")	Expanders - Apply equivalent subjects<br>Search modes - Find all my search terms	Interface - EBSCOhost Research Databases<br>Search Screen - Advanced Search<br>Database - CINAHL with Full Text	Display
S35	(MH "Social Integration")	Expanders - Apply equivalent subjects<br>Search modes - Find all my search terms	Interface - EBSCOhost Research Databases<br>Search Screen - Advanced Search<br>Database - CINAHL with Full Text	Display
S34	(MH "Stigma") OR "stigma*"	Expanders - Apply equivalent subjects<br>Search modes - Find all my search terms	Interface - EBSCOhost Research Databases<br>Search Screen - Advanced Search<br>Database - CINAHL with Full Text	Display
S33	"social exclu*"	Expanders - Apply equivalent subjects<br>Search modes - Find all my search terms	Interface - EBSCOhost Research Databases<br>Search Screen - Advanced Search<br>Database - CINAHL with Full Text	Display
S32	"social depriva*"	Expanders - Apply equivalent subjects<br>Search modes - Find all my search terms	Interface - EBSCOhost Research Databases<br>Search Screen - Advanced Search<br>Database - CINAHL with Full Text	Display
S31	(MH "Social Deprivation")	Expanders - Apply equivalent subjects<br>Search modes - Find all my search terms	Interface - EBSCOhost Research Databases<br>Search Screen - Advanced Search<br>Database - CINAHL with Full Text	Display
S30	(MH "Social Participation") OR "social participa*"	Expanders - Apply equivalent subjects<br>Search modes - Find all my search terms	Interface - EBSCOhost Research Databases<br>Search Screen - Advanced Search	Display

			Database - CINAHL with Full Text	
S29	"social accept*"	Expanders - Apply equivalent subjects<br>Search modes - Find all my search terms	Interface - EBSCOhost Research Databases<br>Search Screen - Advanced Search<br>Database - CINAHL with Full Text	Display
S28	(MH "Social Status")	Expanders - Apply equivalent subjects<br>Search modes - Find all my search terms	Interface - EBSCOhost Research Databases<br>Search Screen - Advanced Search<br>Database - CINAHL with Full Text	Display
S27	(MH "Social Inclusion")	Expanders - Apply equivalent subjects<br>Search modes - Find all my search terms	Interface - EBSCOhost Research Databases<br>Search Screen - Advanced Search<br>Database - CINAHL with Full Text	Display
S26	"alcoholi*"	Expanders - Apply equivalent subjects<br>Search modes - Find all my search terms	Interface - EBSCOhost Research Databases<br>Search Screen - Advanced Search<br>Database - CINAHL with Full Text	Display
S25	(MH "Alcohol Abuse") OR "alcohol abuse"	Expanders - Apply equivalent subjects<br>Search modes - Find all my search terms	Interface - EBSCOhost Research Databases<br>Search Screen - Advanced Search<br>Database - CINAHL with Full Text	Display
S24	"alcohol misuse"	Expanders - Apply equivalent subjects<br>Search modes - Find all my search terms	Interface - EBSCOhost Research Databases<br>Search Screen - Advanced Search<br>Database - CINAHL with Full Text	Display
S23	"alcohol use"	Expanders - Apply equivalent subjects<br>Search modes - Find all my search terms	Interface - EBSCOhost Research Databases<br>Search Screen - Advanced Search<br>Database - CINAHL with Full Text	Display
S22	"substance misuse"	Expanders - Apply equivalent subjects<br>Search modes - Find all my search terms	Interface - EBSCOhost Research Databases<br>Search Screen - Advanced Search<br>Database - CINAHL with Full Text	Display
S21	(MH "Substance Abuse") OR "substance abuse"	Expanders - Apply equivalent subjects<br>Search modes - Find all my search terms	Interface - EBSCOhost Research Databases<br>Search Screen - Advanced Search<br>Database - CINAHL with Full Text	Display
S20	"substance use"	Expanders - Apply equivalent subjects<br>Search modes - Find all my search terms	Interface - EBSCOhost Research Databases<br>Search Screen - Advanced Search	Display

			Database - CINAHL with Full Text	
S19	(MH "Substance Abuse")	Expanders - Apply equivalent subjects<br>Search modes - Find all my search terms	Interface - EBSCOhost Research Databases<br>Search Screen - Advanced Search<br>Database - CINAHL with Full Text	Display
S18	(MH "Substance Use Disorders")	Expanders - Apply equivalent subjects<br>Search modes - Find all my search terms	Interface - EBSCOhost Research Databases<br>Search Screen - Advanced Search<br>Database - CINAHL with Full Text	Display
S17	"psychiatric illness*"	Expanders - Apply equivalent subjects<br>Search modes - Find all my search terms	Interface - EBSCOhost Research Databases<br>Search Screen - Advanced Search<br>Database - CINAHL with Full Text	Display
S16	"psychiatric illness*"	Expanders - Apply equivalent subjects<br>Search modes - Find all my search terms	Interface - EBSCOhost Research Databases<br>Search Screen - Advanced Search<br>Database - CINAHL with Full Text	Display
S15	"psychiatric disorder*"	Expanders - Apply equivalent subjects<br>Search modes - Find all my search terms	Interface - EBSCOhost Research Databases<br>Search Screen - Advanced Search<br>Database - CINAHL with Full Text	Display
S14	"psychiatric disorder"	Expanders - Apply equivalent subjects<br>Search modes - Find all my search terms	Interface - EBSCOhost Research Databases<br>Search Screen - Advanced Search<br>Database - CINAHL with Full Text	Display
S13	(MH "Mental Disorders")	Expanders - Apply equivalent subjects<br>Search modes - Find all my search terms	Interface - EBSCOhost Research Databases<br>Search Screen - Advanced Search<br>Database - CINAHL with Full Text	Display
S12	(MH "Mental Health")	Expanders - Apply equivalent subjects<br>Search modes - Find all my search terms	Interface - EBSCOhost Research Databases<br>Search Screen - Advanced Search<br>Database - CINAHL with Full Text	Display
S11	"mental illness"	Expanders - Apply equivalent subjects<br>Search modes - Find all my search terms	Interface - EBSCOhost Research Databases<br>Search Screen - Advanced Search<br>Database - CINAHL with Full Text	Display
S10	(MH "Mental Health")	Expanders - Apply equivalent subjects<br>Search modes - Find all my search terms	Interface - EBSCOhost Research Databases<br>Search Screen - Advanced Search	Display

			Database - CINAHL with Full Text	
S9	S1 OR S2 OR S3 OR S4 OR S5 OR S6 OR S7 OR S8	Expanders - Apply equivalent subjects Search modes - Find all my search terms	Interface - EBSCOhost Research Databases Search Screen - Advanced Search Database - CINAHL with Full Text	Display
S8	"coexist*"	Expanders - Apply equivalent subjects Search modes - Find all my search terms	Interface - EBSCOhost Research Databases Search Screen - Advanced Search Database - CINAHL with Full Text	Display
S7	"concurrent"	Expanders - Apply equivalent subjects Search modes - Find all my search terms	Interface - EBSCOhost Research Databases Search Screen - Advanced Search Database - CINAHL with Full Text	Display
S6	"comorbid*"	Expanders - Apply equivalent subjects Search modes - Find all my search terms	Interface - EBSCOhost Research Databases Search Screen - Advanced Search Database - CINAHL with Full Text	Display
S5	(MH "Comorbidity")	Expanders - Apply equivalent subjects Search modes - Find all my search terms	Interface - EBSCOhost Research Databases Search Screen - Advanced Search Database - CINAHL with Full Text	Display
S4	"cooccur*"	Expanders - Apply equivalent subjects Search modes - Find all my search terms	Interface - EBSCOhost Research Databases Search Screen - Advanced Search Database - CINAHL with Full Text	Display
S3	"co-occur*"	Expanders - Apply equivalent subjects Search modes - Find all my search terms	Interface - EBSCOhost Research Databases Search Screen - Advanced Search Database - CINAHL with Full Text	Display
S2	(MH "Diagnosis, Dual (Psychiatry)")	Expanders - Apply equivalent subjects Search modes - Find all my search terms	Interface - EBSCOhost Research Databases Search Screen - Advanced Search Database - CINAHL with Full Text	Display
S1	"dual diagnosis"	Expanders - Apply equivalent subjects Search modes - Find all my search terms	Interface - EBSCOhost Research Databases Search Screen - Advanced Search Database - CINAHL with Full Text	Display

Nederst i skjemaet

Focused Search conducted September 2023

APA PsycInfo <1806 to August Week 4 2023>

#	Searches	
1	dual diagnosis/ or "dual diagnosis".ti,ab.	3335
2	(co-occur* or cooccur* or comorbid* or concurrent* or coexist*).ti,ab.	135443
3	comorbidity/	40012
4	or/2-3	146579
5	exp mental disorders/	1035849
6	mental health services/ or exp community mental health services/	47318
7	psychiatric patients/	29522
8	((mental or psychiatric) adj3 (illness* or disorder*)).ti,ab.	153618
9	or/5-8	1121602
10	exp "substance use disorder"/	123335
11	((substance or drug or alcohol) adj3 (abuse or misuse or disorder* or addict* or dependen* or problem*)).ti,ab.	124289
12	((illicit or illegal) adj3 ("alcohol use" or "drug use" or "substance use")).ti,ab.	5162
13	or/10-12	181771
14	1 or (4 and 9 and 13)	21974
15	exp social acceptance/	11381
16	exp social deprivation/	10734
17	social disadvantage/	410
18	social adjustment/	10141
19	exp social identity/	20674
20	exp stigma/	18221
21	((social* or commun*) adj2 (accept* or adjust* or inclusion or includ* or integrat* or interact* or identi* or involv* or reintegrat* or participat* or relations* or skills or support*)).ti,ab.	254546
22	((social* or commun*) adj2 (depriv* or disadvantage* or disintegrat* or isolat* or marginal* or exclud* or withdraw* or challeng*)).ti,ab.	28707
23	citizenship/	6156
24	exp employment status/	32161
25	exp community involvement/	7158
26	exp housing/	11161
27	"recovery (disorders)"/	15130
28	(employ* or unemploy* or educat* or housing or stigma* or recovery or ostrac* or belong*).ti,ab.	960045
29	or/15-28	1197434
30	14 and 29	4427
31	limit 30 to "qualitative (maximizes specificity)"	291
32	limit 31 to yr="2000 -Current"	280
33	limit 32 to all journals	195
34	limit 33 to (danish or english or norwegian or swedish)	185

185 imported to Endnote

Ovid MEDLINE(R) ALL <1946 to September 06, 2023>

#	Searches	
1	"Diagnosis, Dual (Psychiatry)"/ or "dual diagnosis".ti,ab.	4738
2	(co-occur* or cooccur* or comorbid* or concurrent* or coexist*).ti,ab.	538278
3	Comorbidity/	124966
4	or/2-3	610124
5	exp Mental Disorders/	1440525
6	exp mental health services/ or community mental health services/ or social work, psychiatric/	105827
7	((mental or psychiatric) adj3 (illness* or disorder*)).ti,ab.	154238
8	or/5-7	1556360
9	exp Substance-Related Disorders/	311512
10	((substance or drug or alcohol) adj3 (abuse or misuse or disorder* or addict* or dependen* or problem*)).ti,ab.	155624
11	((illicit or illegal) adj3 ("alcohol use" or "drug use" or "substance use")).ti,ab.	6631
12	or/9-11	384120
13	1 or (4 and 8 and 12)	28387
14	exp social structure/	450
15	exp Social Isolation/	25689
16	Social Marginalization/	591
17	social skills/	2698
18	social stigma/	12932
19	social identification/	10098
20	Social Adjustment/	23677
21	((social* or commun*) adj2 (accept* or adjust* or inclusion or includ* or integrat* or interact* or identi* or involv* or reintegrat* or participat* or relations* or skills or support*)).ti,ab.	219416
22	((social* or commun*) adj2 (depriv* or disadvantage* or disintegrat* or isolat* or marginal* or exclud* or withdraw* or challeng*)).ti,ab.	35384
23	exp Employment/	100283
24	community participation/ or community support/	18572
25	housing/ or public housing/	21608
26	(employ* or unemploy* or educat* or housing or stigma* or recovery or ostrac* or belong*).ti,ab.	2317690
27	or/14-26	2610810
28	13 and 27	5493
29	limit 28 to "qualitative (maximizes specificity)"	285
30	limit 29 to yr="2000 -Current"	279
31	limit 30 to (danish or english or norwegian or swedish)	272

272 imported to EndNote

Embase <1980 to 2023 Week 35>

#	Searches	
1	exp dual diagnosis/ or "dual diagnosis".ti,ab.	3487
2	(co-occur* or cooccur* or comorbid* or concurrent* or coexist*).ti,ab.	789709
3	comorbidity/	386837

4	or/2-3	943855
5	exp mental disease/	2551060
6	mental health service/ or exp community mental health service/	62999
7	mental patient/	28174
8	((mental or psychiatric) adj3 (illness* or disorder*)).ti,ab.	203857
9	or/5-8	2607991
10	exp drug dependence/	260101
11	exp drug abuse/	137029
12	((substance or drug or alcohol) adj3 (abuse or misuse or disorder* or addict* or dependen* or problem*)).ti,ab.	212791
13	((illicit or illegal) adj3 ("alcohol use" or "drug use" or "substance use")).ti,ab.	9131
14	or/10-13	450164
15	1 or (4 and 9 and 14)	40610
16	exp social acceptance/	4400
17	exp social isolation/	31584
18	social adaptation/	22230
19	social identity/	514
20	exp social structure/	647811
21	exp social exclusion/	2850
22	social competence/	7715
23	exp social stigma/	15241
24	stigma/	18578
25	((social* or commun*) adj2 (accept* or adjust* or inclusion or includ* or integrat* or interact* or identi* or involv* or reintegrat* or participat* or relations* or skills or support*)).ti,ab.	267713
26	((social* or commun*) adj2 (depriv* or disadvantage* or disintegrat* or isolat* or marginal* or exclud* or withdraw* or challeng*)).ti,ab.	44183
27	employment status/ or neet status/ or unemployment/	46856
28	exp housing/	29710
29	mental health recovery/	728
30	(employ* or unemploy* or educat* or housing or stigma* or recovery or ostrac* or belong*).ti,ab.	2852016
31	or/16-30	3612368
32	15 and 31	9299
33	limit 32 to "qualitative (maximizes specificity)"	337
34	limit 33 to yr="2000 -Current"	333
35	limit 34 to journal	333
36	limit 35 to (danish or english or norwegian or swedish)	321

321 imported to EndNote

Scopus 08.09.2023

(((ABS ((social* OR commun*) W/1 (accept* OR adjust* OR inclusion OR includ* OR integrat* OR interact* OR identi* OR involv* OR reintegrat* OR participat* OR relations* OR skills OR support*))) OR (ABS ((social* OR commun*) W/1 (depriv* OR disadvantage* OR disintegrat* OR isolat* OR marginal* OR exclud* OR withdraw* OR challeng*))) OR (ABS (employ* OR unemploy* OR educat*

OR housing OR stigma* OR recovery OR ostrac* OR belong*))) AND (((ABS (co-occur* OR cooccur* OR comorbid* OR concurrent* OR coexist*)) AND (ABS ((mental OR psychiatric) W/2 (illness* OR disorder*))) AND ((ABS ((substance OR drug OR alcohol) W/2 (abuse OR misuse OR disorder* OR addict* OR dependen* OR problem*))) OR (ABS ((illicit OR illegal) W/2 ("alcohol use" OR "drug use" OR "substance use"))))) OR (ABS ("dual diagnosis"))) AND PUBYEAR > 1999 AND PUBYEAR < 2024 AND PUBYEAR > 1999 AND PUBYEAR < 2024) AND (TITLE (qualitative OR interview* OR "focus group" OR phenomenol* OR "grounded theory" OR narrativ*) AND PUBYEAR > 1999) AND (LIMIT-TO (LANGUAGE , "English") OR LIMIT-TO (LANGUAGE , "Swedish") OR LIMIT-TO (LANGUAGE , "Norwegian") OR LIMIT-TO (LANGUAGE , "Danish")) AND (LIMIT-TO (DOCTYPE , "ar") OR LIMIT-TO (DOCTYPE , "re")) 46 imported to EndNote

Monday, September 11, 2023 7:03:38 AM

CINAHL+ with Full text 11.09.23

#	Query	Limiters/Expanders	Results
S1	(MH "Diagnosis, Dual (Psychiatry)")	Expanders - Apply equivalent subjects Search modes - Find all my search terms	2,725
S2	AB co-occur* or cooccur* or comorbid* or concurrent* or coexist*	Expanders - Apply equivalent subjects Search modes - Find all my search terms	131,513
S3	(MH "Comorbidity")	Expanders - Apply equivalent subjects Search modes - Find all my search terms	71,435
S4	S2 OR S3	Expanders - Apply equivalent subjects Search modes - Find all my search terms	176,811
S5	(MH "Mental Disorders+")	Expanders - Apply equivalent subjects Search modes - Find all my search terms	649,956
S6	(MH "Mental Health Services") OR (MH "Community Mental Health Services") OR (MH "Social Work, Psychiatric")	Expanders - Apply equivalent subjects Search modes - Find all my search terms	47,409
S7	(MH "Psychiatric Patients")	Expanders - Apply equivalent subjects Search modes - Find all my search terms	14,857
S8	AB (mental or psychiatric) N2 (illness* or disorder*)	Expanders - Apply equivalent subjects Search modes - Find all my search terms	52,907
S9	S5 OR S6 OR S7 OR S8	Expanders - Apply equivalent subjects Search modes - Find all my search terms	694,624
S10	(MH "Substance Use Disorders") OR (MH "Substance Abuse+") OR (MH "Substance Dependence+")	Expanders - Apply equivalent subjects Search modes - Find all my search terms	184,935
S11	(substance or drug or alcohol) N2 (abuse or misuse or disorder* or addict* or dependen* or problem*)	Expanders - Apply equivalent subjects Search modes - Find all my search terms	168,769
S12	(illicit or illegal) N2 ("alcohol use" or "drug use" or "substance use")	Expanders - Apply equivalent subjects Search modes - Find all my search terms	3,627
S13	S10 OR S11 OR S12	Expanders - Apply equivalent subjects Search modes - Find all my search terms	240,540
S14	S1 OR (S4 AND S9 AND S13)	Expanders - Apply equivalent subjects Search modes - Find all my search terms	18,832
S15	(MH "Social Structure+")	Expanders - Apply equivalent subjects Search modes - Find all my search terms	622
S16	(MH "Social Isolation+")	Expanders - Apply equivalent subjects Search modes - Find all my search terms	17,679
S17	(MH "Social Skills")	Expanders - Apply equivalent subjects Search modes - Find all my search terms	3,851

S18	(MH "Social Identity")	Expanders - Apply equivalent subjects<br>Search modes - Find all my search terms	10,963
S19	(MH "Social Adjustment")	Expanders - Apply equivalent subjects<br>Search modes - Find all my search terms	4,413
S20	AB (social* or commun*) N1 (accept* or adjust* or inclusion or includ* or integrat* or interact* or identi* or involv* or reintegrat* or participat* or relations* or skills or support*)	Expanders - Apply equivalent subjects<br>Search modes - Find all my search terms	111,701
S21	AB (social* or commun*) N1 (depriv* or disadvantage* or disintegrat* or isolat* or marginal* or exclud* or withdraw* or challeng*)	Expanders - Apply equivalent subjects<br>Search modes - Find all my search terms	15,897
S22	(MH "Employment+")	Expanders - Apply equivalent subjects<br>Search modes - Find all my search terms	53,448
S23	(MH "Community Support")	Expanders - Apply equivalent subjects<br>Search modes - Find all my search terms	242
S24	(MH "Housing") OR (MH "Home Ownership") OR (MH "Public Housing")	Expanders - Apply equivalent subjects<br>Search modes - Find all my search terms	10,952
S25	AB employ* or unemploy* or educat* or housing or stigma* or recovery or ostrac* or belong*	Expanders - Apply equivalent subjects<br>Search modes - Find all my search terms	624,822
S26	S15 OR S16 OR S17 OR S18 OR S19 OR S20 OR S21 OR S22 OR S23 OR S24 OR S25	Expanders - Apply equivalent subjects<br>Search modes - Find all my search terms	768,922
S27	S14 AND S26	Expanders - Apply equivalent subjects<br>Search modes - Find all my search terms	3,439
S28	S14 AND S26	Limiters - Clinical Queries: Qualitative - High Specificity<br>Expanders - Apply equivalent subjects<br>Search modes - Find all my search terms	146
S29	S14 AND S26	Limiters - Published Date: 20000101-20231231; Clinical Queries: Qualitative - High Specificity<br>Expanders - Apply equivalent subjects<br>Search modes - Find all my search terms	144

Nederst i skjemaet

144 imported to EndNote

ORIA 11.09.23
Alle felt inneholder dobbeldiagnose* OR (rus AND psykiatri) OG Alle felt inneholder sosial* OR delta* OG Alle felt inneholder kvalitativ* OR narrativ OR intervju* OR fokusgruppe*
OMFANG: Standard / HINN Biblioteket
37 references, 0 peer reviewed articles

Alle felt inneholder dobbeldiagnose OR rop-lidelse OR rop-pasient OG Alle felt inneholder sosial OR delta OG Alle felt inneholder kvalitativ* OR narrativ OR intervju* OR fokusgruppe*
OMFANG: Standard / Norske fagbibliotek
23 references, 0 peer reviewed articles
